# Supplementary figures and images for: Resolving Anatomical and Functional Structure in Human Brain Organization: Identifying Mesoscale Organization in Weighted Network Representations
Source: PLoS Comput Biol. 2014 Oct 2;10(10):e1003712. doi: 10.1371/journal.pcbi.1003712 (PMC4183375; doi:10.1371/journal.pcbi.1003712)

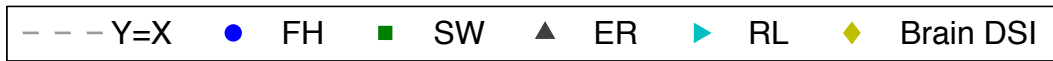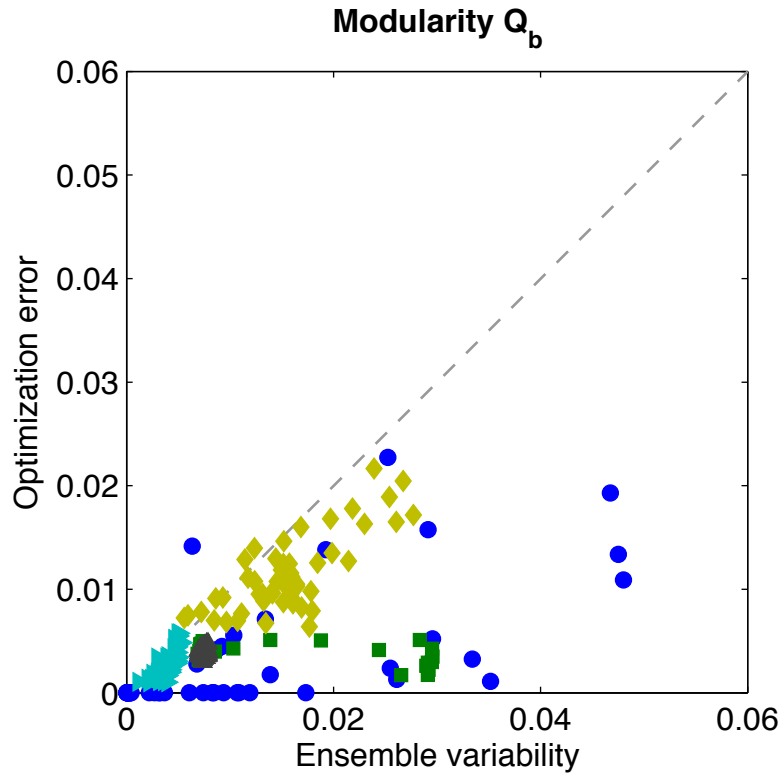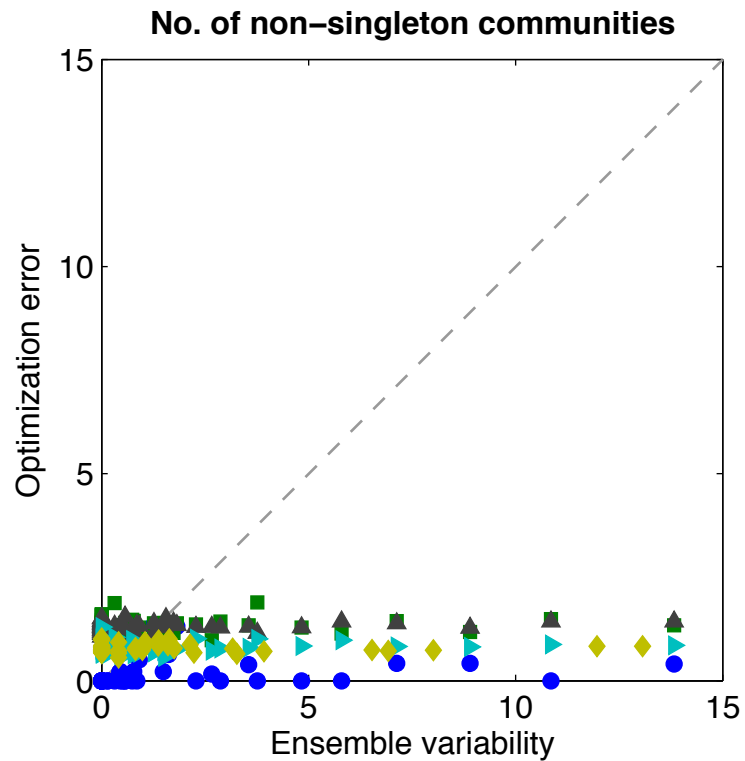

Supplement: Figure S1 — Optimization and realization variance. The optimization variance versus the realization variance in the (A) binary modularity and (B) number of communities for the ensembles of fractal hierarchical (blue), modular small-world (green), Erdös-Rényi (gray), ring lattice (cyan), and DSI brain (gold) networks. The dashed gray line indicates the the line of equivalence between the optimization and randomization variance. (PDF) [file pcbi.1003712.s001.pdf]

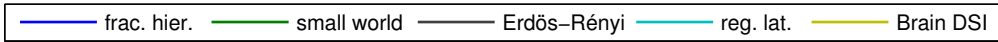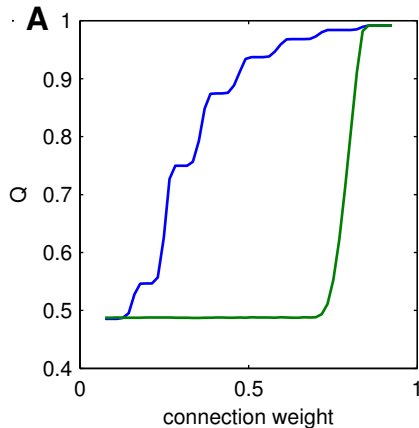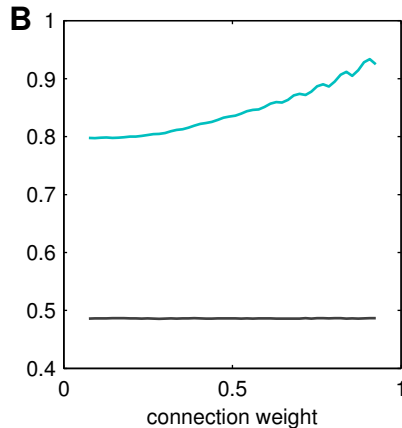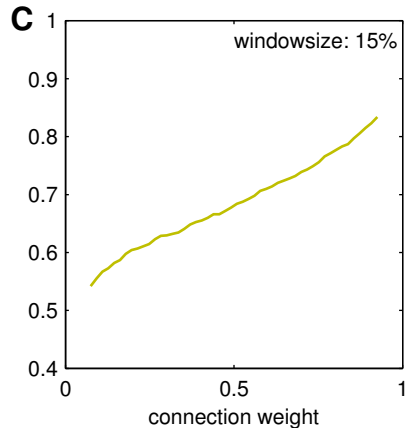

Supplement: Figure S2 — Effect of multiresolution network geometry on community structure. (A) Weighted adjacency matrices depicted for 10% of nodes in the synthetic network models and structural brain networks extracted from DSI data. (B-D) Modularity as a function of the average connection weight of the edges retained in the graph for the (B) fractal hierarchical, small world, (C), Erdös-Rényi, regular lattice, and (D) structural brain network. Window size is 15%. Values of are averaged over 20 optimizations of the binary modularity quality function for each of 50 realizations of a synthetic network model or 6 subjects for the brain DSI network. The standard error of the mean is smaller than the line width. (PDF) [file pcbi.1003712.s002.pdf]

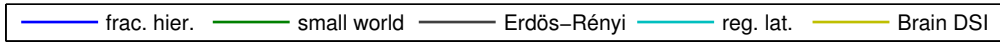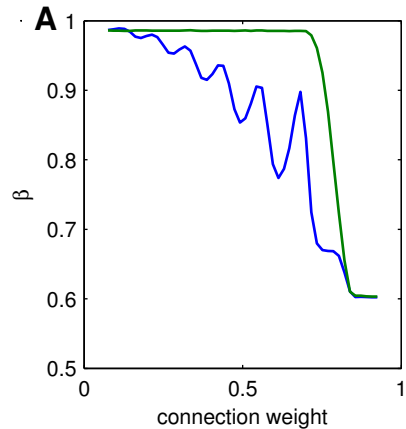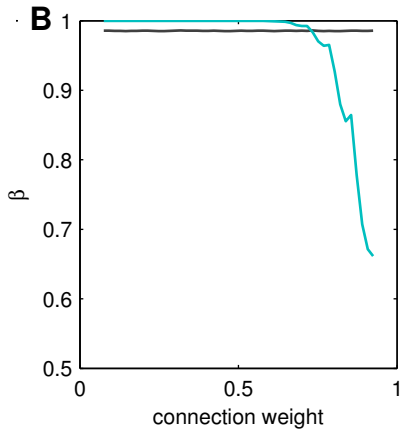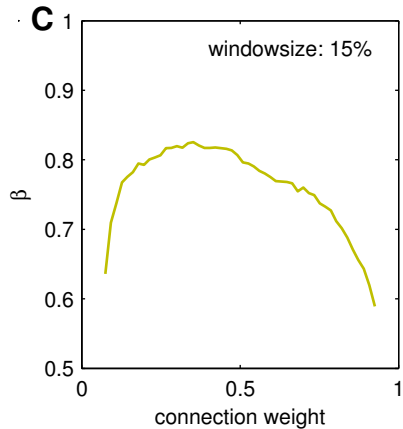

Supplement: Figure S3 — Effect of multiresolution network geometry on bipartite structure. (A) Bipartivity as function of average connection weight of the edges retained in the graph for the (B) fractal hierarchical, small world, (C), Erdös-Rényi, regular lattice, and (D) structural brain network. Window size is 15%. Values of are averaged over 50 realizations of a synthetic network model or 6 subjects for the brain DSI network. The standard error of the mean is smaller than the line width. (PDF) [file pcbi.1003712.s003.pdf]

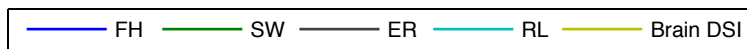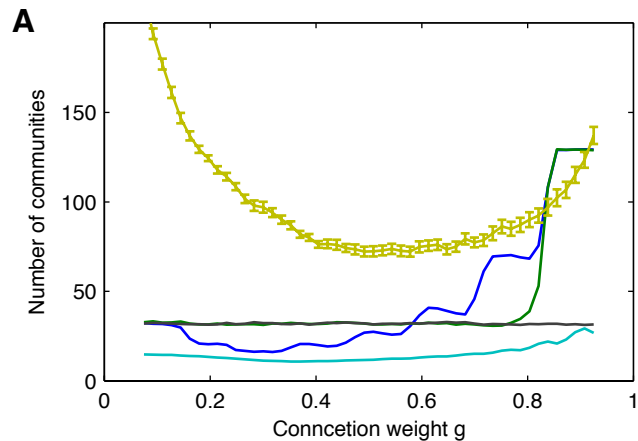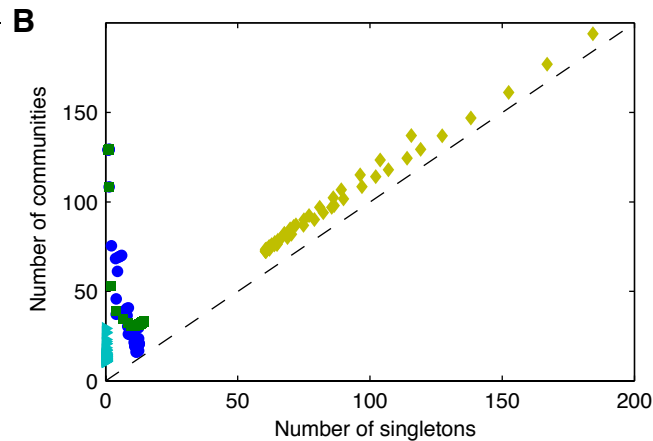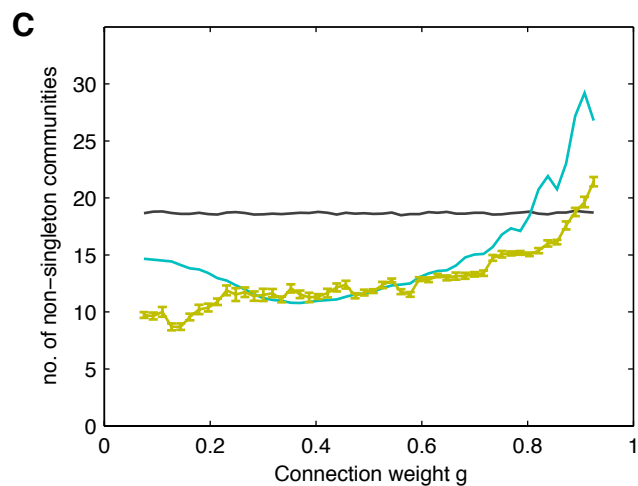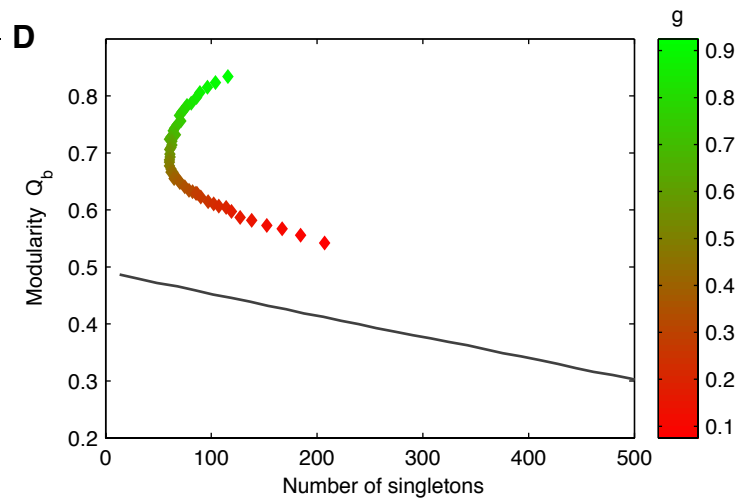

Supplement: Figure S4 — Role of singletons in community number and modularity. (A) Number of communities as a function of average connection weight . (B) Number of singletons versus number of communities. Data points correspond to each graph in the family which captures network organization at different mean connection weights. (C) Number of non-singleton communities as a function of average connection weight . (D) Binary modularity as a function of the number of singletons. The gray line shows the modularity of an Erdös-Rényi random network, when successively disconnecting nodes from the network and randomly adding the same number of connections in the rest of the network. Data points correspond to each graph in the brain DSI family which captures network organization at differen mean connection weights. Color indicates mean connection weight . Window size is 15%. In panels (A) and (C), error bars indicate the standard deviation of the mean; for the model networks this error is smaller than the line width. (PDF) [file pcbi.1003712.s004.pdf]

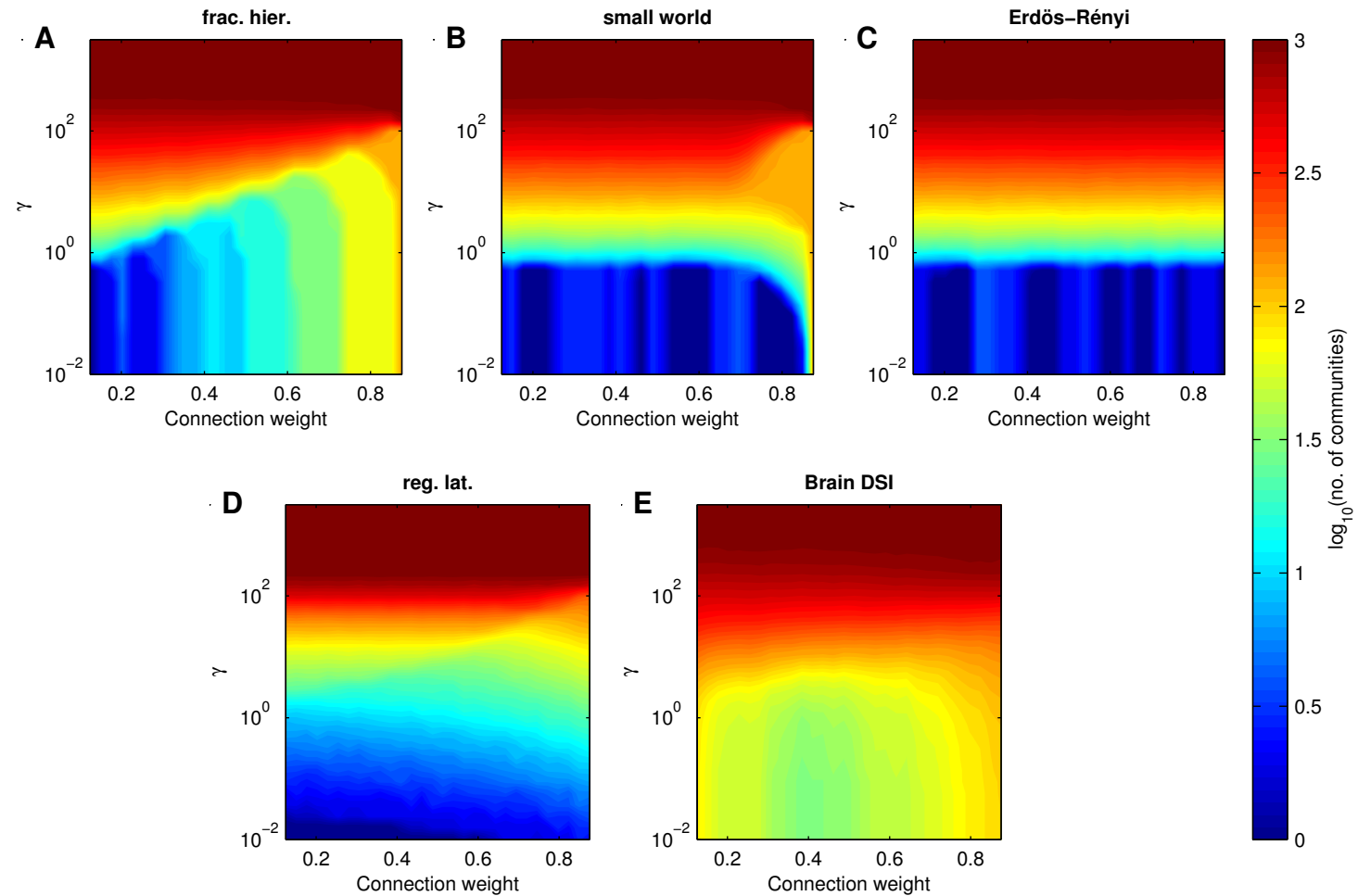

Supplement: Figure S5 — Simultaneously probing structural resolution and network geometry. Colorplots of the total number of communities (singletons and non-singletons) as function of both average connection weight and resolution parameter for the (A) fractal hierarchical, (B) small world, (C) Erdös-Rényi, and (D) regular lattice models and for (E) one representative DSI anatomical network. The window size is 25%. (PDF) [file pcbi.1003712.s005.pdf]
