# Supplementary material for: Resolving Anatomical and Functional Structure in Human Brain Organization: Identifying Mesoscale Organization in Weighted Network Representations
Source: PLoS Comput Biol. 2014 Oct 2;10(10):e1003712. doi: 10.1371/journal.pcbi.1003712 (PMC4183375; doi:10.1371/journal.pcbi.1003712)
Supplement: Table S1 — Description of network ensembles. The number of networks (size) in the ensemble, number of nodes, number of edges given in units of edges for the two types of ensembles studied. Network Type I contains the brain DSI, Erdös-Rényi, Regular Lattice, Fractal Hierarchical, and Small World networks with nodes. Network Type II contains the brain fMRI, Erdös-Rényi, Regular Lattice, Fractal Hierarchical, and Small World networks with nodes. (PDF) [file pcbi.1003712.s006.pdf]

Network Type I	Size of ensemble	Nodes	Edges K $[10^3]$		
Brain DSI	6	1000	14.4	$\pm$	0.5
Erdös-Rényi	50	1000	14.4	$\pm$	0.45
Regular Lattice	50	1000	14.3	$\pm$	0.6
Fractal Hierarchical	50	1000	14.4	$\pm$	0.6
Small World	50	1000	14.4	$\pm$	0.46
Network Type II					
Brain fMRI	29	90	4.05	$\pm$	0
Erdös-Rényi	50	90	0.524	$\pm$	0.025
Regular Lattice	50	90	0.526	$\pm$	0.026
Fractal Hierarchical	50	90	0.526	$\pm$	0.030
Small World	50	90	0.523	$\pm$	0.032

Table 1. Description of Network Ensembles. The number of networks (size) in the ensemble, number of nodes, number of edges K given in units of $[10^3]$ edges for the two types of ensembles studied. Network Type I contains the brain DSI, Erdös-Rényi, Regular Lattice, Fractal Hierarchical, and Small World networks with $N = 1000$ nodes. Network Type II contains the brain fMRI, Erdös-Rényi, Regular Lattice, Fractal Hierarchical, and Small World networks with $N = 90$ nodes.
