# Supplementary material for: Resolving Anatomical and Functional Structure in Human Brain Organization: Identifying Mesoscale Organization in Weighted Network Representations
Source: PLoS Comput Biol. 2014 Oct 2;10(10):e1003712. doi: 10.1371/journal.pcbi.1003712 (PMC4183375; doi:10.1371/journal.pcbi.1003712)
Supplement: Text S1 — Supplementary document including supplementary results on (i) Optimization and Realization Variance, (ii) Effects of Window Size, and (iii) Relationship Between the Number of Communities and Singletons. (PDF) [file pcbi.1003712.s007.pdf]

# Supplementary Online Material for:

# Resolving Structure in Human Brain Organization: Identifying Mesoscale Organization in Weighted Network Representations

Christian Lohse[1], Danielle S. Bassett[2,3,4,*], Kelvin Lim[5] Jean M. Carlson[2]

**1 Kirchhoff Institute for Physics, University of Heidelberg, Germany 69120**

**2 Department of Physics, University of California, Santa Barbara, CA 93106, USA**

**3 Sage Center for the Study of the Mind, University of California, Santa Barbara, CA 93106, USA**

**4 Department of Bioengineering, University of Pennsylvania, Philadelphia, PA 19104, USA**

**5 Department of Psychiatry, University of Minnesota, Minneapolis, MN 55455, USA**

∗ **E-mail: Corresponding dsb@seas.upenn.edu**

In this Supplementary Document, we including the following materials to support the work described in the main manuscript:

- Supplementary Results on Optimization and Realization Variance
- Supplementary Results on the Effects of Window Size
- Supplementary Results on the Relationship Between the Number of Communities and Singletons

# Ensemble Variability and Optimization Error

When comparing two different data sets or models, the interpretation of results can be affected by the relative roles of different sources of variation [1]. In this section, we describe the roles of optimization and realization variance.

**Optimization Variance** Identifying the optimal partition of a network into communities via modularity maximization is NP-hard. We employ a Louvain-like locally greedy heuristic algorithm [2] and perform the optimization multiple times to obtain sets of partitions the capture the representative structure in the network. We define the *optimization variance* to be the standard deviation in network diagnostic values over from these multiple optimizations.

**Realization Variance** Synthetic network models can be used to produce ensembles of binary or weights graphs based on a given construction or growth rule [3] (see Table S1). Similarly, empirical networks from neuroimaging data sets represent ensembles of graphs over different human subjects. We define the *realization variance* to be the standard deviation of network diagnostic values over such multiple empirical or synthetic realizations.

To identify the sensitivity of our results to optimizations and realizations, we determine whether one source of variance is significantly larger in magnitude than another. In general, we find that both types of variance are small in comparison to the quantities of interest such as the modularity or number of communities (see Figure S1). Furthermore, the optimization variance is generally smaller than the realization variance, demonstrating that modularity-based diagnostics are reliable measures of individual variations in mesoscale structures and can be utilized for group comparisons.

# Effects of Window Size

When using the windowed thresholding technique, it is important to understand how the choice of window size affects measured network diagnostic values. Our results are generally robust over a fairly wide range of window sizes between 10% and 25%, showing that this method allows a robust analysis of the graph structure. In this section, we provide results obtained with a window size of 15% to complement the results in the main manuscript obtained with a window size of 25%.

Using a window size of 15%, the MRFs of modularity as a function of connection weight are qualitatively similar to those obtained using a window size of 25% (compare Figure S2 to Figure 2 in the main manuscript). The values of modularity obtained using the smaller window size are in general larger than the values obtained using the larger window size, likely due at least in part to the smaller connection density within each graph of the family. Different window sizes can be used to probe different structures in the underlying network geometry. In the small-world model, we are now able to resolve a second plateau in modularity, corresponding to the elementary groups in the construction process. In the fractal hierarchical network, we are also better able to resolve the lowest hierarchical level. In the regular lattice network, we observe small oscillations corresponding to the different lengths of the connections in the chain lattice.

The MRFs of bipartivity as a function of connection weight obtained using a window of size 15% are also qualitatively similar to those obtained using a window size of 25% (compare Figure S3 to Figure 3 in the main manuscript). The values of bipartivity obtained using the smaller window size are in general larger than the values obtained using the larger window size. Similar to the results observed for modularity MRFs, different window sizes can differentially probe networks with different underlying geometries. In the fractal hierarchical model, we observe oscillations that correspond to the transitions between the hierarchical levels. Low bipartivity values correspond to threshold windows containing both inter- and intra-modular edges; high bipartivity values correspond to larger relative numbers of intermodular edges. In the small world model, we are now able to resolve two plateaus corresponding to (i) the elementary

groups and (ii) the random organization of the long range connections. For the brain network, we observe that $\beta$ now takes values over a much broader interval.

# Number of Communities and Singletons

In the main manuscript, we briefly discussed the fact that when probing community structure across different $\gamma$ values, one can obtain partitions of the network into communities of variable sizes. Singletons are communities composed of a single node. In this section, we examine the role of singletons in two related diagnostics: community number and modularity.

The MRFs of the total number of communities (both singletons and non-singletons) as a function of mean connection weight display different characteristic shapes for different network geometries (see Figure S4A). In the Erdös-Rényi model, the number of communities is constant over the whole range of connection weights, providing a benchmark for model comparison. In the fractal hierarchical network, the different hierarchical levels are evident in the stepwise increases in the community number. In the small-world model, we recover the two distinct structural regimes: (i) the weaker edges display a similar community number to that expected in an Erdös-Rényi network of the same size, and (ii) the strongest edges display the same number of elementary groups as the fractal hierarchical model.

The brain DSI network contains more communities than the synthetic models, especially at either end of the weight spectrum (i.e., strongest and weakest edges). However, unlike the synthetic models, most of the communities in the brain partitions are singletons (see Figure S4B). In fact, we observe an approximately linear relationship between the number of communities and the number of singletons in the brain DSI network that we do not observe in the synthetic network models. This result suggests that the brain displays a relatively simple fragmentation process over different mean connection weights.

The number of non-singleton communities is largest for graphs composed of the strongest edges which we know from the main manuscript display pronounced community structure and smallest for graphs composed of the weakest edges which we know display less pronounced community structure (see Figure S4C). In fact, the value of the binary modularity can be directly mapped to the number of singletons (see Figure S4D). In the human brain, graph composed of weak edges display small values of modularity and a large number of singletons. Graphs composed of medium-weighted edges display middling values of modularity and a small number of singletons. Graphs composed of strong edges display large values of modularity and a large number of singletons.

To determine whether this complex relationship could be expected, we constructed a benchmark Erdös-Rényi model and tuned the number of singletons while retaining a fixed connection density using the following algorithm. We created a set of graphs with a different number of singletons. To create a singleton in a given graph, we removed all edges that emanated from a single node $i$ chosen uniformly at random. Let the number of edges removed be denoted $k_i$. We then add $k_i$ edges to the remaining network, distributing them uniformly at random to all nodes except node $i$. To create many singletons, we iteratively applied the process for creating a single singleton. In this manner, we constructed a set of graphs Erdös-Rényi graph with between 0 and $N/2$ singletons. This process ensures that the number of singletons can be carefully titrated in a graph, the remaining connections still display an Erdös-Rényi topology, and the connection density of the graph is unaltered. The modularity value of this Erdös-Rényi model is approximately linearly related to the number of singletons (see Figure S4D). Note that since the singletons are all disconnected nodes and therefore have a zero contribution to the modularity $Q$, independent of the particular partition. Our approach here is mathematically equivalent to decreasing the number of nodes $N$ while keeping the number of connections $K$ fixed. In this sense one can say that the modularity scales linearly with $N$ over a fairly wide range of the percentage of singletons. This is consistent with the observation of decreasing modularity for higher numbers of connections across the ensemble of benchmark Erdös-Rényi networks.

Thus, the complex relationship between modularity and number of singletons observed in the human

brain is not expected from an benchmark Erdös-Rényi graph. This is perhaps unsurprising given that the graphs in the human brain network family each contain inherently different topologies while the benchmark Erdös-Rényi graphs are constructed to retain the same topology as the number of singletons increases. The results highlight the difficulties in interpreting the actual value of the modularity itself, and instead support efforts in utilizing other diagnostics based on the partition structure [1].

# Supplementary Table and Figure Legends

**Table S1: Description of Network Ensembles.** The number of networks (size) in the ensemble, number of nodes, number of edges $K$ given in units of $[10^3]$ edges for the two types of ensembles studied. Network Type I contains the brain DSI, Erdös-Rényi, Regular Lattice, Fractal Hierarchical, and Small World networks with $N = 1000$ nodes. Network Type II contains the brain fMRI, Erdös-Rényi, Regular Lattice, Fractal Hierarchical, and Small World networks with $N = 90$ nodes.

**Figure S1: Optimization and Realization Variance.** The optimization variance versus the realization variance in the *(A)* binary modularity and *(B)* number of communities for the ensembles of fractal hierarchical (blue), modular small-world (green), Erdös-Rényi (gray), ring lattice (cyan), and DSI brain (gold) networks. The dashed gray line indicates the the line of equivalence between the optimization and randomization variance.

**Figure S2: Effect of Multiresolution Network Geometry on Community Structure** *(A)* Weighted adjacency matrices depicted for 10% of nodes in the synthetic network models and structural brain networks extracted from DSI data. *(B-D)* Modularity $Q_b$ as a function of the average connection weight $g$ of the edges retained in the graph (see Methods section) for the *(B)* fractal hierarchical, small world, *(C)*, Erdös-Rényi, regular lattice, and *(D)* structural brain network. Window size is 15%. Values of $Q_b$ are averaged over 20 optimizations of Equation 1 for each of 50 realizations of a synthetic network model or 6 subjects for the brain DSI network. The standard error of the mean is smaller than the line width.

**Figure S3: Effect of Multiresolution Network Geometry on Bipartite Structure.** *(A)* Bipartivity as function of average connection weight $g$ of the edges retained in the graph (see the Methods section) for the *(B)* fractal hierarchical, small world, *(C)*, Erdös-Rényi, regular lattice, and *(D)* structural brain network. Window size is 15%. Values of $\beta$ are averaged over 50 realizations of a synthetic network model or 6 subjects for the brain DSI network. The standard error of the mean is smaller than the line width.

**Figure S4: Role of Singletons in Community Number and Modularity.** *(A)* Number of communities as a function of average connection weight $g$. *(B)* Number of singletons versus number of communities. Data points correspond to each graph in the family which captures network organization at different mean connection weights. *(C)* Number of non-singleton communities as a function of average connection weight $g$. *(D)* Binary modularity as a function of the number of singletons. The gray line shows the modularity of an Erdös-Rényi random network, when successively disconnecting nodes from the network and randomly adding the same number of connections in the rest of the network. Data points correspond to each graph in the brain DSI family which captures network organization at differen mean connection weights. Color indicates mean connection weight $g$. Window size is 15%. In panels *(A)* and *(C)*, error bars indicate the standard deviation of the mean; for the model networks this error is smaller than the line width.

**Figure S5: Simultaneously Probing Structural Resolution and Network Geometry.** Colorplots of the total number of communities (singletons and non-singletons) as function of both average connection weight $g$ and resolution parameter $\gamma$ for the *(A)* fractal hierarchical, *(B)* small world, *(C)* Erdös-Rényi, and *(D)* regular lattice models and for *(E)* one representative DSI anatomical network. The window size is 25%.

# References

1. Bassett DS, Porter MA, Wymbs NF, Grafton ST, Carlson JM, et al. (2012) Robust detection of dynamic community structure in networks. http://arxivorg/abs/12064358v1 .

2. Jutla IS, Lucas GSJ, Mucha PJ (2011-2012) A generalized louvain method for community detection implemented in matlab. http://netwikiamathuncedu/GenLouvain/GenLouvain .

3. Klimm F, Bassett DS, Carlson JM, Mucha PJ (2013) Resolving structural variability in network models and the brain. arXiv 1306.2893.
